# Supplementary material for: Mental Health during COVID-19 Pandemic among Caregivers of Young Children in Kenya’s Urban Informal Settlements. A Cross-Sectional Telephone Survey
Source: Int J Environ Res Public Health. 2021 Sep 25;18(19):10092. doi: 10.3390/ijerph181910092 (PMC8508326; doi:10.3390/ijerph181910092)
Supplement: Supplementary file 1 [file ijerph-18-10092-s001.zip › ijerph-1301426-supplementary.pdf]

Supplementary Files for Manuscript "Mental Health during COVID-19 Pandemic among Caregivers of Young Children in Kenya's Urban Informal Settlements. A Cross-sectional Telephone Survey"

Supplementary File S1: Patient health questionnaire (English and Swahili Translation)

Over the **last 2 weeks,** have you experienced the following<br><br>*Katika muda* ***wa wiki mbili zilizopita****, je umekuwa na hali zifuatazo*		**Not at all**<br><br>*Haijatokezea kabisa*	**Several days**<br><br>*Siku kadhaa*	**More than half the days**<br>*Zaidi ya nusu ya siku hizo*	**Nearly everyday**<br><br>*Takriban kila siku*
1	Little interest or pleasure in doing things<br>*Kutokuwa na hamu au raha ya kufanya mambo*	0	1	2	3
2	Feeling down, depressed, or hopeless<br>*Kuvunjika moyo, kuhuzunika, au kukosa* matumaini	0	1	2	3
3	Trouble falling asleep, staying asleep, or sleeping too much<br>*Shida ya kupata usingizi, au kuendelea kulala mfululizo, au kulala sana*	0	1	2	3
4	Feeling tired or having little energy<br>*Kujisikia kuchoka au kutokuwa na nguvu*	0	1	2	3
5	Poor appetite or overeating<br>*Kutokuwa na hamu ya kula au kula sana*	0	1	2	3
6	Feeling bad about yourself - or that you're a failure or have let yourself or your family down<br>*Kujihisi vibaya, kujiona umeshindwa kutimiza malengo yako ya maisha, au kuhisi umejidunisha au familia yako*	0	1	2	3
7	Trouble concentrating on things, such as reading the newspaper or watching television or doing other activities<br>*Shida ya kumakinika katika mambo fulani kwa mfano unaposoma gazeti, au kuangalia runinga, au kufanya kazi zingine*	0	1	2	3
8	Moving or speaking so slowly that other people could have noticed (or the opposite) you are restless or moving around a lot more than usual<br>*Kutembea au kuongea polepole sana mpaka watu wakawa wanaona tofauti (au kinyume chake) kwamba hutulii na unahangaika sana kuliko ilivyo kawaida*	0	1	2	3
9	Thoughts that you would be better off dead or of hurting yourself in some way<br>*Kuwaza kuwa ni afadhali zaidi ungekuwa umekufa au ujidhuru kwa namna fulani*	0	1	2	3

Supplementary File S2: General Anxiety Disorder Scale (English and Swahili Translation)

The following questions describe different health state. For each question, please describe how often you have been bothered by the following in the **last two weeks.**

*Maswali yafuatayo yanaeleza hali tofauti za afya. Kwa kila swali, tafadhali nielezee ni kwa kiasi gani umetatizika au kusumbuliwa na hali hizo za afya katika **wiki mbili zilizopita**.*

	Over the **last 2 weeks,** how often have you been bothered by...<br><br>*Katika muda **wa wiki mbili zilizopita**, ni mara ngapi umesumbuliwa na shida ya …*	**Not at all**<br><br>*Haijatokezea kabisa*	**Several days**<br><br>*Siku kadhaa*	**More than half the days**<br><br>*Zaidi ya nusu ya siku hizo*	**Nearly everyday**<br><br>*Takriban kila siku*
1	**In the last 2 weeks,** have you felt nervous, anxious or on edge?<br><br>***Katika wiki mbili zilizopita, Je**, Umejihisi kuwa na hofu, wasiwasi?*	0	1	2	3
2	**In the last 2 weeks,** have you not been able to stop or control worrying**?**<br><br>***Katika wiki mbili zilizopita, Je**, Umeshindwa kujizuia au kudhibiti wasiwasi?*	0	1	2	3
3	**In the last 2 weeks,** have you worried too much about different things?<br><br>***Katika wiki mbili zilizopita, Je**, Umekuwa na wasiwasi mwingi kuhusu mambo tofauti?*	0	1	2	3
4	**In the last 2 weeks,** have you had trouble relaxing?<br><br>**Katika wiki mbili zilizopita, Je**, Umekuwa na wakati mgumu kupumzika?	0	1	2	3
5	**In the last 2 weeks,** have you been so restless that it is hard to sit still?<br><br>***Katika wiki mbili zilizopita, Je**, Umekuwa na hali ya kutotulia kiasi kwamba ikawa ni vigumu kukaa kwa utulivu?*	0	1	2	3
6	**In the last 2 weeks,** have you been easily annoyed or irritable?<br><br>***Katika wiki mbili zilizopita, Je**, Umekasirika au kuudhika kwa haraka?*	0	1	2	3
7	**In the last 2 weeks,** have you felt afraid as if something awful might happen?<br><br>***Katika wiki mbili zilizopita, Je**, Umejihisi kuwa na hofu kana kwamba kitu kibaya kinawezafanyika/tokea?*	0	1	2	3

Supplementary File S3: COVID-19 Anxiety Scale (English and Swahili Translation)

How often have you experienced the following over the **last 2 weeks?**<br><br>*Katika wiki mbili zilizopita ni mara ngapi umehisi hali zifuatazo?*	**Not at all**<br><br>*Haijatokezea kabisa*	**Rare, less than a day or two**<br>*Nadra, kwa siku moja au mbili*	**Several days**<br>*Siku kadhaa*	**More than 7 days**<br>*Zaidi ya siku saba*	**Nearly every day over the last 2 weeks**<br>*Takriban kila siku ya hizo wiki mbili*
1. I felt dizzy, lightheaded, or faint, when I read or listened to news about the coronavirus<br><br>*Nilihisi kisunzi, kichwa kuwa kizito, au kuzimia nilipo somo au kusikiliza habari kuhusu coronavirus.*	0	1	2	3	4
2. I had trouble falling or staying asleep because I was thinking about the coronavirus.<br><br>*Nilikuwa na shida ya kupata usingizi au usingizi kukatika kwasababu nilikuwa nafikiria kuhusu coronavirus.*	0	1	2	3	4
3. I felt paralyzed or frozen when I thought about or was exposed to information about the coronavirus<br><br>*Nilihisi kuishiwa na nguvu nilipofikiria au nilikuwa nikipata habari kuhusu coronavirus.*	0	1	2	3	4
4. I lost interest in eating when I thought about or was exposed to information about the coronavirus.<br><br>*Nilikosa hamu ya kula nilipokuwa nafikiria kuhusu au kupata habari kuhusu coronavirus.*	0	1	2	3	4
5. I felt nauseous or had stomach problems when I thought about or was exposed to information about the coronavirus.<br><br>*Nilihisi kichefachefu au shida ya tumbo nilipokuwa nifikiria kuhusu au kupata habari kuhusu coronavirus.*	0	1	2	3	4

Supplementary File S4: A summary of participant's socio-demographic characteristic stratified by study site/informal settlement.

Characteristics	Dagoretti	Mathare	Bangladesh	P-value
**Age**				
<30	239 (60.35)	109 (61.89)	164 (61.89)	0.914
30 and above	157 (39.65)	101 (38.11)	101 (38.11)	
**Sex**				
Female	373 (94.19)	167 (94.35)	256 (96.60)	0.344
Male	23 (5.81)	10 (5.65)	9 (3.40)	
**Marital status**				
Married or cohabiting	281 (70.96)	121 (68.36)	175 (66.04)	0.060
Single	70 (17.68)	30 (16.95)	38 (14.34)	
Separated/Divorced/Widowed	45 (11.360	26 (14.69)	52 (19.62)	
**Education Level**				
Primary and below	138 (34.85)	77 (43.50)	158 (59.62)	**<0.001**
Primary School	200 (50.51)	84 (47.46)	93 (35.09)	
Tertiary	58 (14.65)	16 (9.04)	14 (5.28)	
**Occupation**				
Formal	22 (5.56)	6 (3.39)	14 (5.28)	0.178
Informal	167 (42.17)	84 (47.46)	135 (50.94)	
Unemployed	207 (52.27)	87 (49.15)	116 (43.77)	
**Household asset index**				
Mean (SD)	2.90 (1.25)	2.33 91.03)	2.08 (1.03)	**<0.001**
**Pregnancy**				
Not pregnant	264 (66.67)	127 (71.75)	170 (64.15)	0.247
Pregnant	132 (33.33)	50 (28.25)	95 (35.85)	
**No. of children**				
0-3	296 (81.77)	132 (82.50)	189 (75.60)	0.114
>3	66 (18.23)	28 (17.50)	61 (24.40)	

*P-value based on chi-square test of association for categorical variables and one-way ANOVA analysis continuous variables. NOTES: ANOVA - Analysis of Variance

Supplementary File S5a: Prevalence of depressive symptoms, COVID-19 anxiety, and general anxiety

	**Yes** n (%)	**No** n (%)
Depressive symptoms	288 (34.08)	557 (65.92)
COVID-19 anxiety	118 (13.96)	727 (86.04)
General anxiety	167 (19.76)	678 (80.24)

Supplementary File S5b: Categorization of depressive symptoms and general anxiety scores

	**PHQ-9** n (%)	**GAD-7** n (%)
None	305 (36.09)	379 (44.85)
Mild	252 (29.82)	299 (35.38)
Moderate	168 (19.88)	100 (11.83)
Severe	120 (14.20)	67 (7.93)

Supplementary File S6: Univariate analysis of relationship between socio-demographic factors, COVID-19 related consequences and psychosocial aspects and participant's mental health scores

	PHQ-9		CAS		GAD-7	
**Characteristics**	Odds ratio (95% C.I)	P-value	Odds ratio (95% C.I)	P-value	Odds ratio (95% C.I)	P-value
**Age**						
<30	1	-	1	-		
30 and above	1.27 (0.95-1.68)	0.106	1.09 (0.73-1.62)	0.676	1.13 (0.80-1.60)	0.481
**Sex**						
Female	1	-	1	-	1	-
Male	1.36 (0.73-2.52)	0.328	0.44 (0.13-1.43)	0.172	1.38 (0.68-2.79)	0.372
**Marital status**						
Married or cohabiting	1	-	1	-	1	-
Single	0.96 (0.63-1.47)	0.847	0.73 (0.39-1.35)	0.317	0.75 (0.43-1.28)	0.291
Separated/Divorced/Widowed	1.78 (1.20-2.63)	**0.004**	0.95 (0.54-1.67)	0.866	1.35 (0.85-2.14)	0.198
**Education Level**						
Tertiary	1	-	1	-	1	-
Primary and below School	1.51 (0.92-2.49)	0.107	2.30 (1.01-5.20)	**0.047**	1.07 (0.60-1.92)	0.821
Secondary	1.13 (0.68-1.87)	0.643	1.80 (0.79-4.12)	0.163	1.06 (0.59-1.90)	0.847
**Occupation**						
Unemployed	1	-	1	-	1	-
Formal	0.84 (0.42-1.69)	0.623	0.34 (0.08-1.45)	0.146	1.73 (0.85-3.53)	0.133
Informal	1.21 (0.90-1.62)	0.199	1.32 (0.89-1.9)	0.164	1.07 (0.75-1.52)	0.706
**Household asset index**	0.74 (0.65-0.84)	**<0.001**	0.82 (0.69-0.98)	**0.031**	0.86 (0.74-1.00)	**0.048**
**Pregnancy**						
Not pregnant	1	-	1	-	1	-
Pregnant	1.27 (0.94-1.72)	0.114	1.05 (0.70-1.59)	0.803	0.94 (0.65-1.35)	0.721
**No. of children**						
0-3	1	-	1	-	1	-
>3	0.99 (0.69-1.43)	0.960	1.34 (0.84-2.15))	0.211	1.54 (1.02-2.32)	**0.038**
**Experienced violence (COVID period)**						
No	1	-	1	-	1	-
Yes	2.86 (1.89-4.31)	**<0.001**	2.11 (1.29-3.48)	**0.003**	2.12 (1.35-3.32)	**0.001**
**Perceived COVID-19 threat as a serious threat**						

No	1	-	1	**-**	1	-
Yes	1.92 (0.94-3.92)	0.075	7.67 (1.05-56.15)	**0.045**	3.69 (1.13-12.03)	**0.031**
**Experienced discrimination (COVID period)**						
No	1	-	1	**-**	1	**-**
Yes	2.92 (2.00-4.29)	**<0.001**	2.55 (1.62-4.03)	**<0.001**	3.24 (2.16-4.86)	**<0.001**
**Experienced job loss (COVID period)**						
Not affected or affected to a less extent	1	-	1	**-**	1	**-**
Very much affected	3.96 (2.37-6.60)	**<0.001**	2.40 (1.22-4.73)	**0.012**	1.78 (1.05-3.03)	**0.033**
**Loss of income generation (COVID period)**						
No	1	-	1	**-**	1	**-**
Yes	2.30 (1.46-3.64)	**<0.001**	2.13 (1.11-4.11)	**0.024**	1.86 (1.07-3.21)	**0.027**
**Ability to pay utilities affected (COVID period)**						
Not affected or affected to a less extent	1	**-**	1	**-**	1	**-**
Very much affected	1.13 (0.76-1.68)	0.535	0.71 (0.43-1.16)	0.166	0.94 (0.60-1.49)	0.802
**Ability to repay loans affected (COVID period)**						
Not affected or affected to a less extent	1	**-**	1	**-**	1	**-**
Very much affected	1.02 (0.71-1.47)	0.916	0.85 (0.53-1.37)	0.503	0.92 (0.59-1.42)	0.700
**Ability to meet basic childcare affected (COVID period)**						
Not affected or affected to a less extent	1	**-**	1	**-**	1	**-**
Very much affected	2.00 (1.40-2.84)	**<0.001**	1.43 (0.89-2.30)	0.138	1.74 (1.14-2.67)	**0.011**
**Movement outside households affected (COVID period)**						
Not affected or affected to a less extent	1	**-**	1	-	1	**-**
Very much affected	1.47 (1.10-1.97)	**0.010**	1.70 (1.12-2.57)	**0.012**	1.59 (1.12-2.27)	**0.010**
**Family interaction affected (COVID period)**						
Not affected or affected to a less extent	1	**-**	1	-	1	**-**
Very much affected	1.66 (1.21-2.26)	**0.001**	1.56 (1.01-2.42)	**0.045**	1.73 (1.18-2.54)	**0.005**
**Survey study site**						
Dagoretti	1	**-**	1	-	1	**-**
Mathare	1.14 (0.78-1.68)	0.497	1.07 (0.59-1.93)	0.831	0.85 (0.53-1.39)	0.521
Bangladesh	2.07 (1.49-2.86)	**<0.001**	2.88 (1.86-4.46)	**<0.001**	1.67 (1.14-2.43)	**0.008**

*Statistical significance (P-value<0.05). Notes: PHQ-9 – patient health questionnaire; SD-standard deviation
